# Gene Co-Expression Network Modular Analysis Reveals Altered Immune Mechanisms in HIV-HAND

**DOI:** 10.3390/brainsci12101378

**Published:** 2022-10-12

**Authors:** Maria Cristina Petralia, Ferdinando Nicoletti, Lyubka Tancheva, Reni Kalfin, Paolo Fagone, Katia Mangano

**Affiliations:** 1 Department of Clinical and Experimental Medicine, University of Messina, 98122 Messina, Italy; 2 Department of Biomedical and Biotechnological Sciences, University of Catania, Via S. Sofia 89, 95123 Catania, Italy; 3 Institute of Neurobiology, Bulgarian Academy of Sciences, Acad. G. Bonchev St., Block 23, 1113 Sofia, Bulgaria; 4 Department of Healthcare, South-West University “Neofit Rilski”, Ivan Mihailov St. 66, 2700 Blagoevgrad, Bulgaria

**Keywords:** HIV, HIV-HAND, HIV-HAD, HIVE neurocognitive impairment, immune response, National NeuroAIDS Tissue Consortium

## Abstract

Although the introduction of HAART has completely changed the natural course of HIV infection, the number of chronic forms of HIV-associated neurocognitive disorder (HAND) has risen. It is estimated that up to half of subjects undergoing HAART therapy exhibit mild cognitive impairments. In the current study, we apply the gene co-expression network modular analysis, a well-established system biology approach, to the gene expression profiles of cases from the National NeuroAIDS Tissue Consortium (NNTC). We observed a negative enrichment for genes associated with the control of immune responses and putatively regulated by the transcription factors IRF8 and SPI1 and by both type I and II interferons. Our study provides evidence of altered immune responses, which are likely associated with the occurrence of HAND in the absence of HIV encephalitis (HIVE).

## 1. Introduction

Since the introduction of HAART, the number of chronic forms of HIV-associated neurocognitive disorder (HAND) has risen. It is estimated that up to half of subjects undergoing HAART therapy exhibit mild cognitive impairments [1], while approximately 10% of HIV-infected patients show neuropathological evidence of HIVE (HIV encephalitis) [2]. Hence, since HAND is considerably more prevalent than HIVE, the pathophysiology mechanisms of HAND need to be better characterized [3]. Despite efficient peripheral viral control, HIV persists in the brain due to the low CNS bioavailability of antiretrovirals and the accumulation of resistance mutations in the CNS. Importantly, HIV RNA load in the central nervous system (CNS) is associated with neurological manifestations [4].

Gelman and colleagues [5] previously showed that samples of the central nervous system of HIV individuals with HAND as compared with those with both HAND and HIVE are characterized by distinctive transcriptomic profiles, indicating that there could be different etiological pathways leading to HAND [5]. In particular, HIVE with HAND was characterized with high brain viral load, with an upregulation of the inflammatory pathways and a downregulation of neuronal transcripts, mostly in the frontal neocortex. On the other hand, HAND without HIVE was associated with a low brain HIV burden without either inflammatory response or downregulation of transcripts in frontal neocortical neurons.

In the current study, we apply the gene co-expression network modular analysis, a well-established system biology approach, to the gene expression profiles of cases from the National NeuroAIDS Tissue Consortium (NNTC), which included samples from three brain regions: white matter, basal ganglia, and frontal cortex. By assessing gene co-expression patterns, this methodology allows a more systematic and global interpretation of gene expression data, identifying “modules” that likely comprise functionally related genes and overcoming the problem of multiple comparisons. 

We observed, in the frontal cortex, a negative enrichment for genes associated with the control of immune responses and putatively regulated by the transcription factors IRF8 and SPI1 and by both type I and II interferons. Overall, our study provides evidence of altered immune responses, which are likely associated with the occurrence of HAND in the absence of HIVE.

## 2. Materials and Methods

### 2.1. Dataset Selection

For the current study, the GSE35864 dataset [5] was retrieved from the Gene Expression Omnibus database (http://www.ncbi.nlm.nih.gov/geo/, accessed on 2 September 2022). The dataset included 24 cases divided into 4 groups: uninfected with no neuropathological abnormalities (n = 6), HIV + neurocognitively normal with no neuropathology (n = 6), HAND with no HIVE or substantial neuropathological defect (n = 7), and HIV with HAND and HIVE (n = 5). For all the patients, data were obtained from the frontal cortex (Brodmann area 9), basal ganglia (head of the caudate nucleus), and white matter (deep frontal lobe). Clinical data are provided in Table 1. The gene expression dataset was generated using the Affymetrix GeneChip Human Genome U133 Plus 2.0 arrays. In the current study, in accordance with Levine et al. (2013) [3], 21 of the 24 cases were used, while WM-A6-06 and all three samples from subjects B5, B6, and D5 were removed.

### 2.2. Co-Expression Modular Network Analysis

The gene co-expression network analysis was performed using the CEMiTool software. [6] Briefly, CEMiTool uses an unsupervised filtering method based on the inverse gamma distribution to select the genes used in the analyses and employs a soft-thresholding power β to determine a similarity criterion between pairs of genes. The genes are then separated into modules using the Dynamic Tree Cut package. When different sample groups are specified, the genes from co-expression modules are treated as gene sets, and the z-score normalized expression of the samples within each group is treated as rankings. The results determine whether the activity of a module is altered across different phenotypes. 

The interactions between the genes in each co-expression module were constructed using the GeneMANIA database [7]. The top 10 network hubs (i.e., the genes with the highest number of connections) are highlighted in the graph. 

The biological functions related to modules were annotated using the over-representation analysis (ORA), which is based on the hypergeometric test to determine the most significant module functions. The MSigDB (Molecular Signatures Database) [8] C2 collection was used for the ORA analysis.

### 2.3. Evaluation of the Involvement of Different Brain Cell Types in the Different Co-Expression Modules 

In order to determine the enrichment of the different brain cell types in the co-expression modules, we obtained consensus brain cell gene signatures for astrocytes, microglia, neurons, and oligodendrocytes from a paper by McKenzie et al. [9]. The significance of the over-representation of the brain-cell-type-specific genes was obtained using the chi-square test. The representation factor was defined as the number of overlapping genes divided by the expected number of overlapping genes drawn from the two groups. 

### 2.4. Pathway Analysis

For pathway analysis, we used the gene set enrichment analysis (GSEA) [10] and ORA [11] implemented in the WebGestalt (WEB-based Gene SeT AnaLysis Toolkit) software [12]. The analyses were performed using the genesets from the KEGG (Kyoto Encyclopedia of Genes and Genomes) database [13]. Multiple testing adjustment was performed, and significance was assessed using the False Discovery Rate (FDR) in a GSEA algorithm. An FDR < 0.05 was used as threshold for statistical significance.

### 2.5. Gene Module Regulation

For the identification of the transcription factors (TFs) and ligands that could regulate the expression of the genes of interest, the EnrichR web-based utility was employed. EnrichR is a comprehensive database that integrates gene function, ontology, pathways, and statistical tools able to functionally analyze genomewide data from sequencing, proteomics, and gene expression studies [14].

## 3. Results

### 3.1. Gene Co-Expression Network Modular Analysis

**Figure 1:**
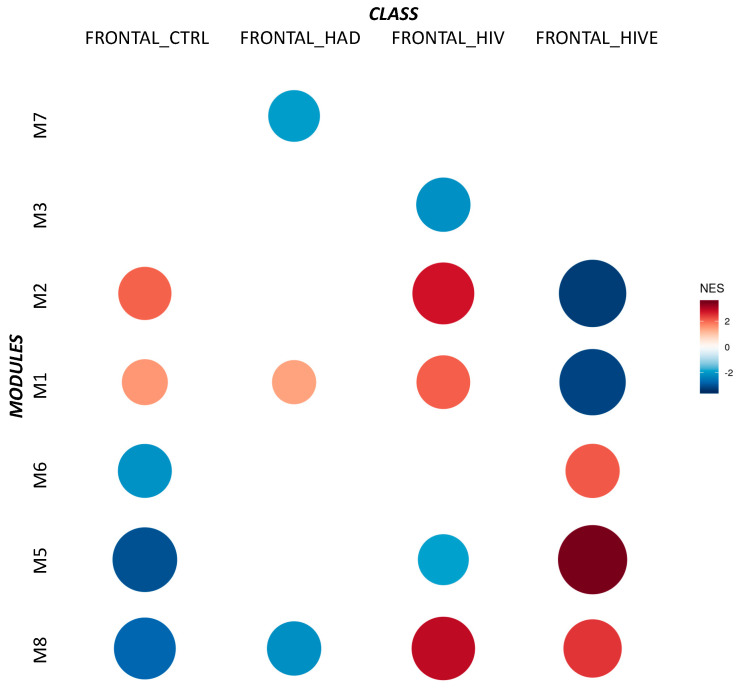
Gene co-expression network modular analysis. Modules enriched by the co-expression network modular analysis of the GSE35864 dataset. The heatmap is color- and size-coded based on the NES (normalized enrichment score) and statistical significance.

**Figure 2:**
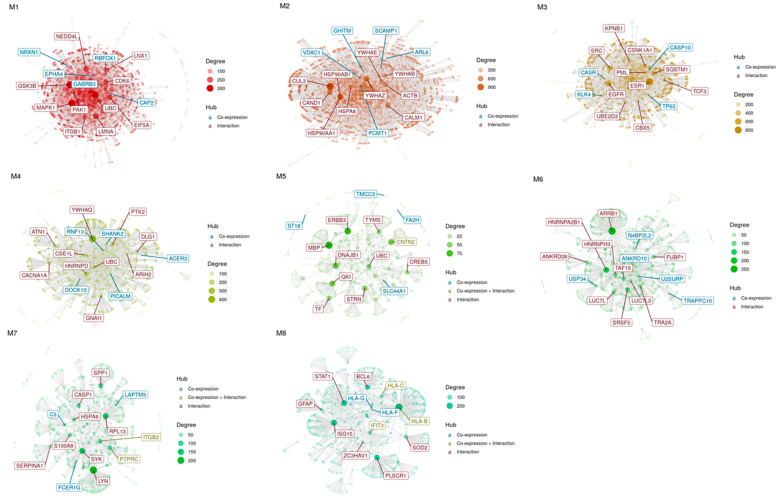
Gene co-expression network modular analysis. PPI network for each of the modules identified in the co-Expression network modular analysis of the GSE35864 dataset. Hub genes are indicated in red.

**Figure 3:**
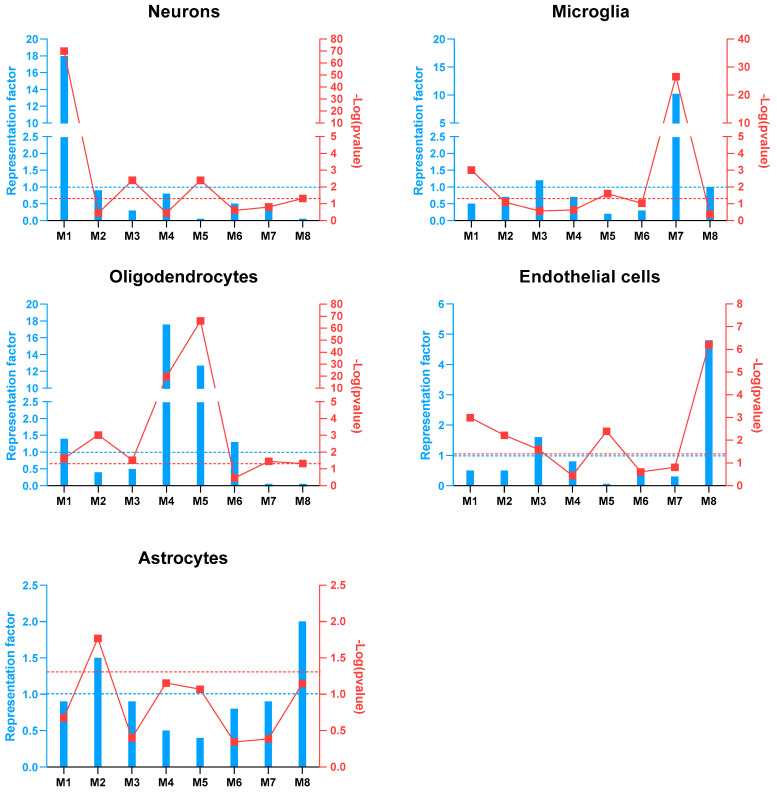
Gene co-expression network modular analysis. Over-representation analysis for the brain cell populations in each of the modules identified in the co-expression network modular analysis of the GSE35864 dataset.

To identify the co-expression networks associated with HIV-HAD, CEMiTool analysis was carried out to disclose highly correlated gene modules. The analysis was performed separately for the samples from the frontal cortex (Brodmann area 9), basal ganglia (head of the caudate nucleus), and white matter (deep frontal lobe). No significant enriched modules were found either for the basal ganglia or for the white matter samples, while eight modules were enriched among the frontal cortex samples (Figure 1, Table 2). Module (M) 1 included 544 genes, M2 307 genes, M3 200 genes, M4 157, M5 87 genes, M6 62 genes, M7 52 genes, and M8 47 genes. Figure 2 shows the PPI network of the genes belonging to each module, highlighting the hub genes (Figure 2). In particular, the hub genes for M1 were NRXN1, CAP2, GABRB3, RBFOX1, and EPHA4; for M2, GHITM, SCAMP1, ARL6, VDAC1, and PCMT1; for M3, TP63, ZNF503-AS2, CASR, KLK4, and CASP10; for M4, DOCK10, SHANK2, RNF13, ACER3, and PICALM; for M5, FA2H, CNTN2, TMCC3, SLC44A1, and ST18; for M6, N4BP2L2, U2SURP, TRAPPC10, USP34, and ANKRD10; for M7, C3, ITGB2, LAPTM5, FCER1G, and PTPRC; for M8, HLA-B, HLA-C, HLA-F, HLA-G, and IFIT3 (Figure 2). Enrichment analysis for the different brain cell populations showed that neurons were enriched among the M1 genes; oligodendrocytes among the M1, M4, and M5 genes; microglia among the M7 genes; endothelial cells among the M3 and M8 genes; and astrocytes among the M2 genes (Figure 3). 

**Figure 4:**
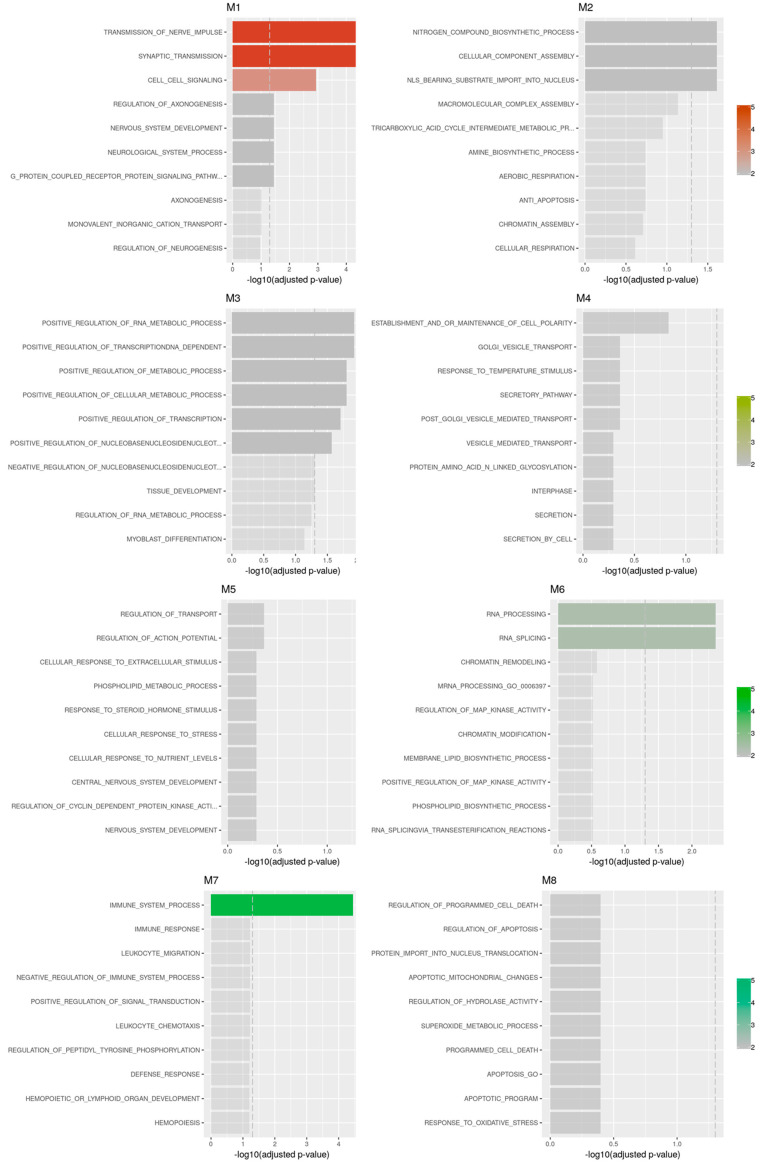
Biological interpretation of the gene co-expression modules obtained from the analysis of the GSE35864 dataset.

ORA analysis identified that the most biological processed enriched for the different modules were respectively, for M1, “transmission of nerve impulse”; for M2, “nitrogen compound biosynthetic process”; for M3, “positive regulation of RNA metabolic process”; for M4, establishment and/or maintenance of cell polarity”; for M5, “regulation of transport”; for M6, “RNA processing”; for M7, “immune system process”; no statistical significance was reached for M8 (Figure 4).

### 3.2. Gene Co-Expression Network Modular Analysis of HIV-HAND

As shown in Figure 1 and Table 2, frontal samples from HIV-HAD patients were characterized by a negative enrichment of Module 7 (NES = −1.93; adj. *p*-value = 0.00082).

The list of genes belonging to M7 includes: ADGRB3, AHI1, AIF1, ALOX5, ARHGDIB, ASPH, C1QC, C3, CALHM6, CASP1, CD163, CD74, CTSC, CTSS, CX3CR1, DOCK8, FCER1G, FGL2, FYB1, GLUL, GOLGA8A, GUSBP1, HLA-DPA1, HLA-DRA, HSPA6, ITGB2, LAPTM5, LST1, LYN, MAF, MS4A4A, MS4A6A, MS4A7, NAPSB, NKAIN3-IT1, P2RY12, PDZD2, PTPRC, RNASET2, RPL13, S100A8, SAMSN1, SCIN, SERPINA1, SLC16A3, SLC4A4, SPP1, STAB1, SUGP2, SYK, TNFSF10, and TNRC6B.

We also observed a significant enrichment of M1 (NES = 1.41; adj. *p*-value = 0.0025), and M8 (NES = −2.13; adj. *p*-value = 0.00082).

On the other hand, frontal samples from uninfected subjects showed a significant enrichment for M1 (NES = −1.53; adj. *p*-value = 0.00043), M2 (NES = −2.04; adj. *p*-value = 0.00043), M5 (NES = −3.02; adj. *p*-value = 0.00043), and M8 (NES = −2.76; adj. *p*-value = 0.00043); HIV-infected samples were characterized by M1 (NES = −2.08; adj. *p*-value = 0.00144), M2 (NES = 2.77; adj. *p*-value = 0.00119), M3 (NES = −2.13; adj. *p*-value = 0.00066), and M8 (NES = 2.91; adj. *p*-value = 0.00082); HIVE showed significant enrichment for M1 (NES = −3.18; adj. *p*-value = 0.00036), M2 (NES = −3.27; adj. *p*-value = 0.00036), M5 (NES = −3.46; adj. *p*-value = 0.00036), and M7 (NES = −2.46; adj. *p*-value = 0.00036).

**Figure 5:**
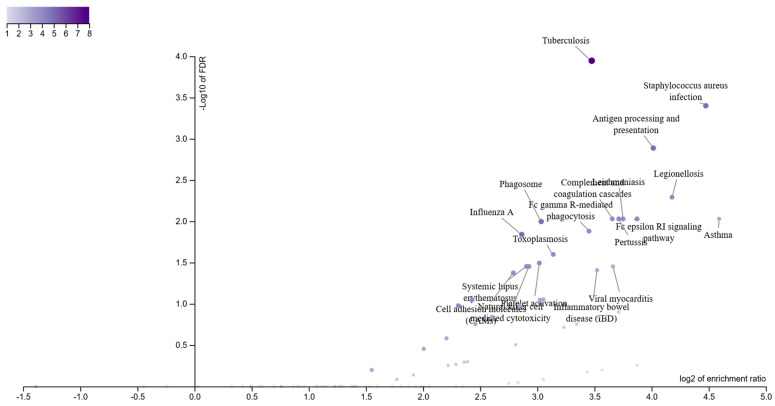
Biological interpretation of Module 7. Over-representation analysis (ORA) for the genes belonging to Module 7, performed using the KEGG database.

**Figure 6:**
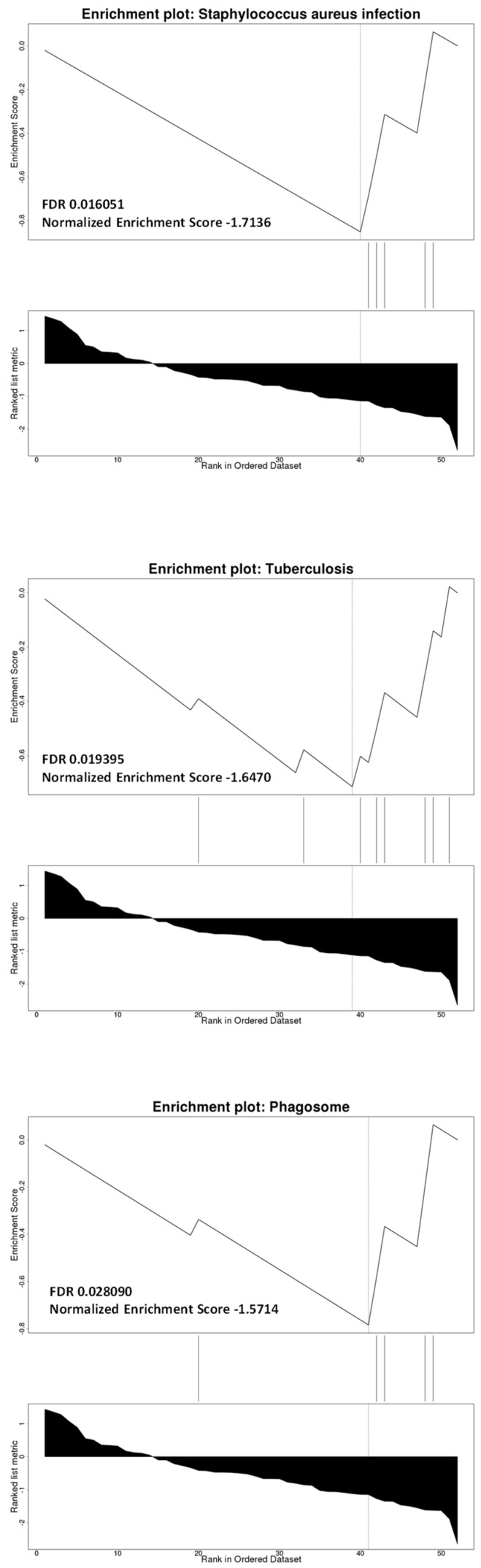
Biological interpretation of Module 7. GSEA analysis for the genes belonging to Module 7 performed using the KEGG database. The significant pathways are shown.

To gain further insight on the biological significance of Module 7 in HIV-HAND, we performed ORA and GSEA analysis using the KEGG database. As shown in Figure 5 and Figure 6, ORA and GSEA analysis concordantly identified three biological processes to be enriched, that is, “*Staphylococcus aureus* infection”, “tuberculosis”, and “phagosome” (Figure 5 and Figure 6). Additional pathways identified by ORA analysis were “antigen processing and presentation”, “complement and coagulation cascades”, and “Fc gamma R-mediated phagocytosis” (Figure 5).

**Figure 7:**
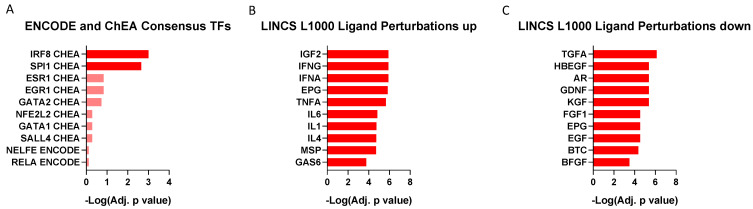
Regulation of Module 7 genes. (**A**) Putative transcription factors involved in the regulation of the expression of the genes belonging to Module 7, (**B**) ligand perturbation analysis showing the factor able to upregulate the expression of the genes belonging to Module 7, (**C**) ligand perturbation analysis showing the factor able to downregulate the expression of the genes belonging to Module 7.

The putative transcription factors regulating the genes belonging to M7 were IRF8 (known to transcribe: CD74, CASP1, HLA-DRA, AIF1, CTSS) and SPI1 (known to transcribe: LYN, AHI1, SYK, DOCK8, LST1, ITGB2, LAPTM5, RPL13, AIF1, SAMSN1, CTSS) (Figure 7A). We also investigated potential ligands able to upregulate the expression of the M7 genes, in particular IGF2, IFNG, and IFNA (Figure 7B), and, conversely, ligands able to downregulate them, including TGFA and HBEGF (Figure 7C).

## 4. Discussion

Despite the efficacy of HAART, in HIV infected patients, there is still a high prevalence of mild and chronic forms of neurocognitive impairment (NCI) [15]. Indeed, HIV invades the CNS and remains in the brain throughout the infection, as the brain represents a sanctuary, due to an ineffective penetration of antiretroviral drugs [16]. NCI is associated with synaptodendritic degeneration [17], and it is believed that chronic neuroinflammation drives neurodegeneration [18,19]. However, the pathogenic mechanisms underlying HAND remain unclear.

To better understand the pathogenetic processes underlying HIV-associated neurological complications, we employed the gene co-expression network modular analysis as an alternative for the analysis of transcriptome data, which usually involves the evaluation of the differentially expressed genes among the compared populations and the determination of their ontological categories. By using the current approach, genes that show correlated expression, and therefore likely work in coordinated biologically related networks, are used to examine important traits of disease pathogenesis.

Our data show that in the frontal cortex of patients with HIV-HAND, there is a significant down-enrichment of a gene module associated with the immune response (i.e., Module 7), which mainly involves the microglial cells. This observation is in line with data generated by Sanna et al. (2017) [20], who showed evidence of the induction of both type I and II IFN-related pathways in HIV patients with HIVE, but not in HAND patients [20], and in partial accordance with another study, in which IFN-gamma was not closely correlated with NCI [21]. Accordingly, our ligand perturbation analysis showed a significant representation of genes in Module 7 that are upregulated by IFNG and IFNA. The data on the role of the IFNs in NCI are often contradictory. Indeed, while IFN-α transgenic mice showed only mild deficit in an egocentric spatial working memory test [22], administration of IFN-β in mice significantly impaired spatial memory [23].

On the other hand, the ligand perturbation analysis identified several factors that could be associated with a downregulation of Module 7 genes, such as members of the EGF family, including TGFA, HBEGF, and EGF, but also FGF1 and GDNF. Fibroblast growth factors (FGFs) are involved in brain development and in neuroprotection. Altered CSF levels of FGF have been previously observed in amyotrophic lateral sclerosis [24] and Alzheimer’s disease [25]. Few data are, however, available on NCI in the course of HIV [26], reporting a correlation between lower FGF-1 levels and NCI [26].

Novel therapeutic strategies aiming at counter-regulating the expression levels of the genes associated with HIV-HAND, by targeting the above-mentioned ligands, could therefore be envisaged.

Our data also identified IRF8 and SPI1, the transcription factors mainly involved in the regulation of Module 7 genes. SPI1 (a.k.a. PU.1) is a member of the Ets family of transcription factors, which is expressed solely in the hematopoietic lineage. PU.1 represents a key regulator of hematopoiesis, and mice bearing homozygous mutations in the DNA binding domain die to septicemia, soon after birth [27]. A complex cross-talk between HIV-1 Tat and the transcription factor PU.1 has recently been described [27]. In particular, in myeloid cells, PU.1 was found to act on the HIV-1 LTR promoter, inhibiting HIV gene expression, including Tat, while Tat inhibits the activity of PU.1, antagonizing the inhibitory effect of PU.1 on long terminal repeat (LTR)-based transcription [27].

IRF8, the gene encoding for the interferon regulatory factor-8 (IRF-8), was previously found to be hypermethylated in HIV-infected individuals with cognitive impairment, as compared with those without NCI, suggesting a potential role for this transcription factor in HIV-related cognitive dysfunction [28].

Finally, the observation of the significant negative enrichment for Module 1 (pertaining to neuronal involvement) in the frontal cortex of patients with HIVE, but only the reduced NES for HAND without HIVE samples, as compared with HIV patients without HAND, suggests that NCI may be accompanied by a progression of functional abnormalities involving the frontal cortex neurons.

While the findings of the present study are interesting, there are some caveats to mention. First, the sample population included in the study is very small, which negatively impacts on the statistical power of our analysis. Second, our gene co-expression network modular analysis was able to retrieve statistically enriched modules only on the frontal cortex samples, but not in the basal ganglia and in the white matter of the deep frontal lobe. Hence, we may not exclude that other pathogenetic pathways could be implicated in these latter regions. Third, the association between the transcriptomic data and the clinical data could be biased by variables, such as time-to-sample collection, adherence to HAART, and causes of death.

Despite these limitations, we believe that we have provided here a system biology interpretation of brain transcriptome data derived from HIV-HAND individuals. Our results, built upon previously published data, expand the knowledge on HIV neuropathogenesis and add novel findings that warrant further investigation. Our data may also have translational relevance for early diagnosis and preventive treatment for HAND development in HIV-infected individuals by monitoring these and eventually related identified analytes and considering tailored therapeutic interventions aimed at stimulating defective immunomodulatory and antiviral response. Recent preclinical and clinical evidence suggest that direct delivery of these peptides/proteins at the level of the CNS may be feasible [29].

## Figures and Tables

**Table 1:** Clinical characteristics of the patients’ cohort.

Patients	Global Impairment Score	White Matter HIV-1 RNA (log10 copies/g)	Neostriatum HIV-1 RNA (log10 copies/g)	Frontal Cortex HIV-1 RNA (log10 copies/g)	Age at Death (Years ± SD)	Sex
CTRL		0	0	0	50.0 ± 10.4	Male = 5; Female = 1
HIV	4.0 ± 1.4	3.53 ± 0.69	3.84 ± 0.50	3.65 ± 0.74	49.5 ± 8.4	Male = 6; Female = 0
HAND	6.9 ± 1.1	3.85 ± 2.54	3.51 ± 1.37	3.63 ± 1.12	43.7 ± 9.8	Male = 7; Female = 0
HIVE	7.5 ± 2.2	7.40 ± 1.80	7.27 ± 0.84	6.16 ± 1.34	42.8 ± 8.9	Male = 5; Female = 0

**Table 2:** Results for the gene co-expression network modular analysis performed on the GSE35864 dataset.

Pathway	FRONTAL_CTRL: Adjusted *p*-Value	FRONTAL_CTRL: NES	FRONTAL_HAND: Adjusted *p*-Value	FRONTAL_HAND: NES	FRONTAL_HIV: Adjusted *p*-Value	FRONTAL_HIV: NES	FRONTAL:HIVE: Adjusted *p*-Value	FRONTAL:HIVE: NES
M1	0.00043	1.53	0.0025	1.41	0.00144	2.08	0.00036	−3.18
M2	0.00043	2.04	1	−0.52	0.00119	2.77	0.00036	−3.27
M3	0.99909	−0.63	0.50783	1.09	0.00066	−2.13	0.48958	1.06
M4	0.05481	−1.33	1	−0.58	0.06453	1.27	0.93217	0.78
M5	0.00043	−3.02	1	−0.57	0.00066	−1.88	0.00036	3.46
M6	0.00043	−2.1	0.50783	1.1	0.19262	−1.19	0.00036	2.12
M7	0.24902	1.17	0.00082	−1.93	0.1325	1.26	0.75452	−0.88
M8	0.00043	−2.76	0.00082	−2.13	0.00082	2.91	0.00036	2.46

## Data Availability

The data used for the present manuscript are freely available in the NCBI Gene Expression Omnibus (GEO) database (http://www.ncbi.nlm.nih.gov/geo/, accessed on 2 September 2022).
